# Effect of local infiltration analgesia, peripheral nerve blocks, general and spinal anesthesia on early functional recovery and pain control in total knee arthroplasty

**DOI:** 10.1186/s12891-018-2154-z

**Published:** 2018-07-18

**Authors:** M. T. Berninger, J. Friederichs, W. Leidinger, P. Augat, V. Bühren, C. Fulghum, W. Reng

**Affiliations:** 1 endogap, Joint Replacement Institute, Garmisch-Partenkirchen Medical Center, Auenstr. 6, 82467 Garmisch-Partenkirchen, Germany; 2 Department of Trauma Surgery, BG Trauma Center Murnau, Prof.-Küntscher Str. 8, 82418 Murnau, Germany; 3 Department of Anesthesiology and Intensive Care, Garmisch-Partenkirchen Medical Center, Auenstr. 6, 82467 Garmisch-Partenkirchen, Germany; 4 Institute of Biomechanics, BG Trauma Center Murnau, Prof.-Küntscher Str. 8, 82418 Murnau, Germany; 5 Institute of Biomechanics, Paracelsus Medical University, Strubergasse 21, 5020 Salzburg, Austria

**Keywords:** Local infiltration analgesia, Femoral nerve block, Total knee arthroplasty, Epidural catheter, General anesthesia, Spinal anesthesia

## Abstract

**Background:**

Postoperative pain control and enhanced mobilization, muscle strength and range of motion following total knee arthroplasty (TKA) are pivotal requisites to optimize rehabilitation and early recovery. The aim of the study was to analyze the effect of local infiltration analgesia (LIA), peripheral nerve blocks, general and spinal anesthesia on early functional recovery and pain control in primary total knee arthroplasty.

**Methods:**

Between January 2016 until August 2016, 280 patients underwent primary TKA and were subdivided into four groups according to their concomitant pain and anesthetic procedure with catheter-based techniques of femoral and sciatic nerve block (group GA&FNB, *n* = 81) or epidural catheter (group SP&EPI, *n* = 51) in combination with general anesthesia or spinal anesthesia, respectively, and LIA combined with general anesthesia (group GA&LIA, *n* = 86) or spinal anesthesia (group SP&LIA, *n* = 61). Outcome parameters focused on the evaluation of pain (NRS scores), mobilization, muscle strength and range of motion up to 7 days postoperatively. The cumulative consumption of (rescue) pain medication was analyzed.

**Results:**

Pain relief was similar in all groups, while the use of opioid medication was significantly lower (up to 58%) in combination with spinal anesthesia, especially in SP&EPI. The LIA groups, in contrast, revealed significant higher mobilization (up to 26%) and muscle strength (up to 20%) in the early postoperative period. No analgesic technique-related or surgery-related complications occurred within the first 7 days. Due to insufficient pain relief, 8.4% of the patients in the catheter-based groups and 12.2% in the LIA groups resulted in a change of the anesthetics pain management.

**Conclusions:**

The LIA technique offers a safe and effective treatment option concerning early functional recovery and pain control in TKA. Significant advantages were shown for mobilization and muscle strength in the early postoperative period while pain relief was comparable within the groups.

## Background

Postoperative pain after total knee arthroplasty (TKA) is still a challenging issue. For a successful outcome, pain control following TKA is essential to achieve early mobilization, good functional outcome, optimal rehabilitation and enhanced recovery [1]. Therefore, the primary goals of peri- and postoperative analgesic treatment are to reduce pain and immobilization and to minimize opioid requirements and opioid-related adverse events. Enhancing these outcomes has a potential beneficial influence on patient morbidity and satisfaction, the degree of required postoperative care, as well as economic perspectives.

There is a considerable debate on the optimal form of postoperative analgesia in total knee replacement [2]. Several peri- and postoperative pain control strategies have been established in the last years including peripheral nerve blocks, epidural analgesia as well as local infiltration analgesia (LIA) and (systemic) opioids [1].

Regional anesthesia techniques are widely used to deliver intraoperative analgesia and to minimize postoperative pain after TKA [1, 3]. In general, femoral nerve blocks (FNB) are the preferred method for postoperative pain reduction by blocking the femoral nerve, the lateral femoral cutaneous nerve and branches of the obturator nerve; however, pain - especially in the popliteal space - may not be completely under control [4]. Therefore, the combination of FNB with a sciatic nerve block (SNB) seems to be a suitable option to optimize pain management [5, 6]. In terms of pain control, epidural anesthesia in knee surgery did not show any advantages compared to regional analgesia such as FNB [3]. However, it may result in a higher side-effect profile including severe neuraxial complication compared to FNB [7, 8].

As an alternative method for postoperative pain control after TKA, LIA around the soft tissues of replaced knee joints was introduced by Kerr and Kohan in 2008 with an increasing interest in recent years [9]. The analgesic agents are intraoperatively administered by the surgeon to block pain conduction directly. Thereby, systemic side effects associated with postoperative analgesics as well as additional invasive procedures (e.g. administration of analgesic catheters) for pain management are minimized. Moreover, compared to peripheral nerve blockades LIA lacks the possible disadvantage of motor impairment and quadriceps weakness, the risk of nerve injury or limitation of patient ability to ambulate in the immediate postoperative period [10].

Several studies successfully demonstrated significant pain relief and reduced opioid requirements in the early postoperative period by use of LIA compared to placebo [11–13] or (peripheral) analgesic catheters [14, 15]. Data comparing LIA with continuous epidural analgesia are limited and favor LIA over continuous epidural analgesia, particularly due to the side effects and early mobilization [16–18]. The comparability of the results of previous trials is, however, restricted by different anesthesia procedures (general versus spinal anesthesia) and systemic pain medication.

In literature, there is no study analyzing the effect of LIA, (peripheral) catheter-based techniques and their combination with general or spinal anesthesia in one patient collective on pain control, mobilization, muscle strength and range of motion for up to 7 days postoperatively. The hypothesis of this study was that each anesthetic procedure directly influences the immediate postoperative status of the patient in terms of pain control, mobilization, muscle strength and range of motion. Therefore, the aim of the study was to analyze the effect of femoral nerve block and general anesthesia (group GA&FNB), epidural catheter and spinal anesthesia (group SP&EPI), LIA combined with general anesthesia (group GA&LIA) and spinal anesthesia (group SP&LIA), respectively, on early functional recovery and pain control in primary TKA.

## Methods

### Patients

During the period from January 2016 until August 2016, 300 patients underwent primary TKA in a single center for alloplasty. Finally, a total of 280 patients were eligible for the study having received general or spinal anesthesia in combination with a FNB, epidural catheter or LIA. The inclusion criterion was primary TKA for osteoarthritis. Patients were excluded if they had a primary constrained prosthesis, secondary arthritis due to rheumatoid arthritis or trauma, osteonecrosis or revision surgery. This study was performed in conformity with the Declaration of Helsinki and was approved by the Ethics Committee of the Bavarian State Chamber of Physicians (ID: 2017–109). Patient demographics, surgery details, and clinical data are shown in Table 1. Pain medication and use of opioids prior to the surgery in daily life were elaborated from the patients’ medical history.

**Table 1:** Patients demographics and clinical data

	GA&FNB	SP&EPI	GA&LIA	SP&LIA
Patients	*n* = 82	n = 51	n = 86	n = 61
Gender	f = 48; m = 34	f = 31; m = 20	f = 52; m = 34	f = 38; m = 23
Age	67 ± 11.4	71 ± 9.3	68 ± 10.2	70 ± 9.0
Chronic pain patient	n = 5(*6.1%*) with opioid: *n* = 2	n = 2 (*3.9%*) with opioid: *n* = 1	n = 5(*5.8%*) with opioid: *n* = 3	n = 2 (*3.3%*) with opioid: n = 1
Piritramide	OP: *n* = 34 (*41.4%*): 10.0 mg ± 5.4	OP: n = 6 (*11.8%*): 6.9 mg ± 1.9	OP: *n* = 53 (*61.6%*): 9.9 mg ± 4.4	OP: n = 6 (*10.0%*): 7.5 mg ± 0.0
	Day 1: *n* = 7 (*8.5%*): 8.6 mg ± 2.8	Day 1: n = 1 (*2.0%*): 7.5 mg ± 0.0	Day 1: n = 6 (*7.0%*): 9.0 mg ± 3.7	Day 1: n = 6 (*10.0%*): 8.8 mg ± 3.1
	Day 2: n = 2 (*2.4%*): 7.5 mg ± 0.0	Day 2: n = 1 (*2.0%*): 7.5 mg ± 0.0	Day 2: n = 1 (*1.2%*): 9.0 mg ± 3.7	Day 2: n = 2 (*3.3%*): 10.8 mg ± 4.6
Salvage pain management	n = 4 (4.9%) →PCIA: *n* = 4	n = 7 (13.7%) →PCIA: n = 1 →3in1: n = 6	n = 9 (10.5%) →3in1: *n* = 9	n = 9 (14.8%) →3in1: n = 8 →PCIA: n = 4
LIA	–	–	160 ml	160 ml
Dexamethasone	–	–	16.4 mg ± 3.1	15.7 mg ± 3.1

Patients were retrospectively divided into 4 groups according to their received anesthetic and peri-and postoperative analgesic procedure as follows:

Group GA&FNB: General anesthesia + FNB/SNB *n* = 81.

Group SP&EPI: Spinal anesthesia + epidural catheter *n* = 51.

Group GA&LIA: General anesthesia + LIA *n* = 86.

Group SP&LIA: Spinal anesthesia + LIA *n* = 61.

### Anesthetic techniques

After induction of general anesthesia, patients allocated to GA&FNB had a FNB catheter inserted with real-time monitored ultrasound imaging. A total of 20 ml of 0.1% ropivacaine was injected around the femoral nerve; additionally ultrasound-guided sciatic nerve block with 20 ml of 0.1% ropivacaine was established as single shot block. Postoperatively, 0.2% ropivacaine was continuously infused at the rate of 3 ml/h for 3 days through the femoral catheter.

In SP&EPI, a catheter was preoperatively sited at the cranial lumbar vertebrae combined with a spinal anesthesia (1 ml of 0.5% bupivacaine and 10 μg sufentanil in the subarachnoid space) in a single needle technique. After recovery from spinal anesthesia under the level L3, an initial 10 ml bolus containing 0.5% bupivacaine, 0.6 μg/ml sufentanil and saline was introduced. Thereafter, patients were self-medicated with a bolus of 4 ml via a patient-controlled epidural anesthesia (PCEA) system with a lockout of 20 min. PCEA was discontinued three days after surgery.

### Local infiltration anesthesia (LIA)

For the patients in the LIA groups, the surgeons undertook periarticular injection of local anesthetic during surgery. The injection technique used was similar to the technique described by Kerr and Kohan [9]. However, the infiltration used in this study only consisted of 160 ml of 0.2% ropivacaine without any additional components. At the beginning of the anesthesia, dexamethasone (0,2 mg/kg body weight) was injected intravenously. All infiltration was done using 25-ml syringes and 10-cm-long 19-G spinal needles. The LIA solution was administered after completion of all femoral and tibial osteotomy steps, immediately before cement fixation of the tibial component. The LIA solution was systematically injected into the tissues around the knee joint according to a standardized protocol: in the medial and lateral tibial and femoral periosteum as well as medial and lateral posterior articular capsule, and in the subcutaneous tissue, in the Hoffa fat pad and finally intraarticularly after capsular suture.

### Surgery

All surgeries were performed by three senior surgeons. Intraoperatively, single-shot cefazolin 2 g (or clindamycin 600 mg in case of incompatibility of penicillin) for infection prophylaxis was given to all patients. The surgeries were performed with a standard midline vertical incision and medial parapatellar approach. A tourniquet was inflated to 250 mmHg at the beginning of the surgery and deflated after removal of the surgical dressings. In all cases, the LCS**®** complete Knee System (DePuy Synthes, Warsaw, IN, USA) was used; the tibial component was fixed with cement. Bone resections and implant insertion were conducted according to the manufacturers manual. The patella was generally not resurfaced; however, patella osteophytes were removed and circular patella denervation was regularly performed.

### Postoperative pain management and care

Postoperative management was identical in all groups. After surgery, every patient was given peripheral pain medication (WHO grade I, e.g. paracetamol, metamizole, ibuprofen or diclofenac) for about 2 weeks to relieve pain and low molecular weight heparins subcutaneously for about 2 weeks to prevent deep vein thrombosis. The cumulative doses of rescue analgesia (hydromorphone p.o. or piritramide i.v.) were also registered.

Postoperative physiotherapy was started immediately after surgery in a progressive manner and continued daily. A specially trained pain service regularly visited all patients twice a day for the first four postoperative days.

### Outcome measures

Self-reported pain scores in terms of numeric rating scores (NRS) at rest and with activity (0 = no pain; 10 = worst pain) from day of surgery until postoperative day 4 were collected and analyzed. For evaluation of functional outcomes, grade of mobilization ranging from values of 1 to 6 according to our institutional grading system of mobilization was analyzed: 1 = bedridden, 2 = sitting, 3 = standing, 4 = walking in room, 5 = walking on the floor, 6 = walking stairs. Furthermore, muscle strength according to the British medical research council (M0/5-M5/5) and passive range of motion (degrees of extension and flexion) were examined. Functional outcomes of mobilization, muscle strength and range of motion were documented daily from pre-operative day until postoperative day 7, respectively. The patients’ medical files were also studied for potential analgesic technique-related or surgery-related complications within the first 7 days, such as rates of neurologic events, cardiovascular events, falls, knee joint infections, prosthesis loosening, or revision surgery. All data were collected from the patients´ medical records and nurses´ observational charts.

### Data analysis

Statistical analysis was performed with SPSS statistical software 20.0 (SPSS for Windows, ver. 20.0; SPSS, Chicago, IL, USA). Descriptive statistics were calculated for all variables of interest. Continuous measures such as age were summarized using means and standard deviations whereas categorical measures were summarized using counts and percentages.

The Kruskal-Wallis test was used for analysis of one nominal variable and one ranked variable. In a further detailed analysis, post-hoc comparisons of factor-level combinations were conducted by use of Mann-Whitney-U test, depending on previous (overall) significance testing. In this explorative study, no adjustment of an alpha-error level was conducted.

## Results

Baseline characteristics of patients were comparable among all groups (Table 1). About 6% of patients who received general anesthesia (GA&FNB and GA&LIA) compared to 3.5% of patients in the spinal anesthesia groups (SP&EPI and SP&LIA) suffered from chronic pain previously.

Pain exacerbation after surgery due to insufficient pain relief (NRS > 7) with the current anesthetic technique led to another analgesic technique. In GA&FNB, 4.9% (*n* = 4) patients received a patient-controlled intravenous analgesia (PCIA) with an initial bolus of 4 mg piritramide followed by an optional bolus of 2 mg piritramide with a lockout of 10 min. In the other groups, most anesthetic techniques with insufficient pain relief were converted to a secondary application of a FNB. These patients were excluded from outcome measurements. No analgesic technique-related or surgery-related complication occurred in any group within the first postoperative 7 days. At day of surgery, the demand for piritramide was significantly higher (52% vs. 11%; *p* < 0.05) in groups with general anesthesia compared to groups with spinal anesthesia, while it was not significantly different between each other anymore at the first postoperative day (*p* = 0.77). All LIA patients received 160 ml of the LIA injection with 16.4 mg ± 3.1 (group GA&LIA) and 15.7 mg ± 3.1 (group SP&LIA) dexamethasone, respectively.

### Pain

**Fig. 1:**
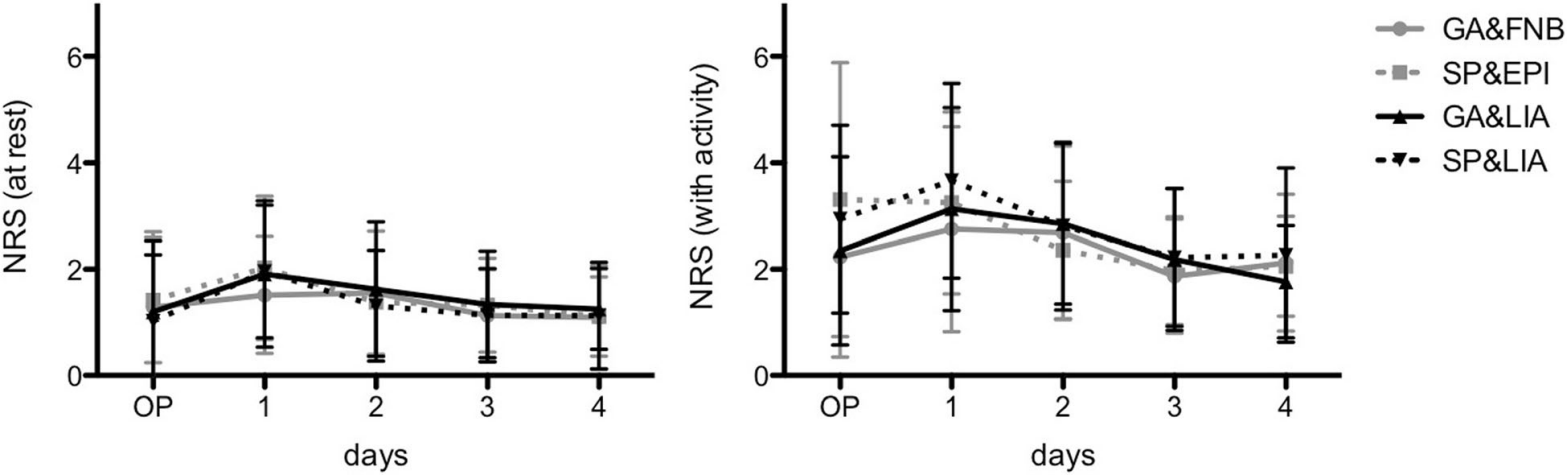
Numeric Rating Scores at rest (left) and with activity (right) are presented for the day of surgery (OP) and postoperative days 1 to 4

The NRS scores (Fig. 1) at rest, although slightly increased at postoperative day 1 (*p* = 0.182), did not show any significant differences at any time (*p* > 0.05). The development of the NRS scores with activity was comparable among groups (p > 0.05); only both groups with spinal anesthesia demonstrated slightly but non-significantly increased pain values at the day of surgery (*p* = 0.132).

**Fig. 2:**
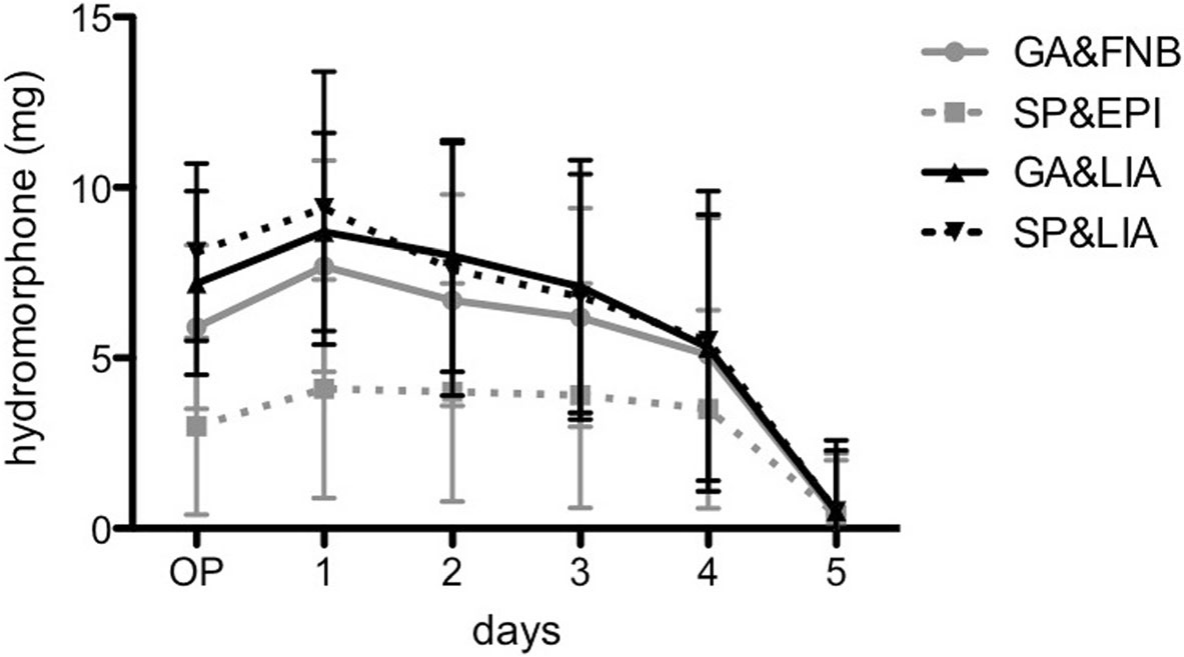
The cumulative dose of hydromorphone (in mg) for all groups at day of surgery until postoperative days 4 is shown

At the day of surgery as well as at postoperative days 1, 2 and 3, the dose of hydromorphone was on average 42 to 58% lower in SP&EPI compared to all other groups (*p* < 0.001; Fig. 2). Additionally, at the day of surgery (OP) both catheter-based groups showed an on average 42% lower dose of hydromorphone compared to the LIA groups (p < 0.001); afterwards, these differences were no longer significant (p > 0.05). In concordance with the indicated increase of the NRS scores at day 1, the dose of hydromorphone seemed to slightly increase, too, in order to gradually fall afterwards.

### Mobilization

**Fig. 3:**
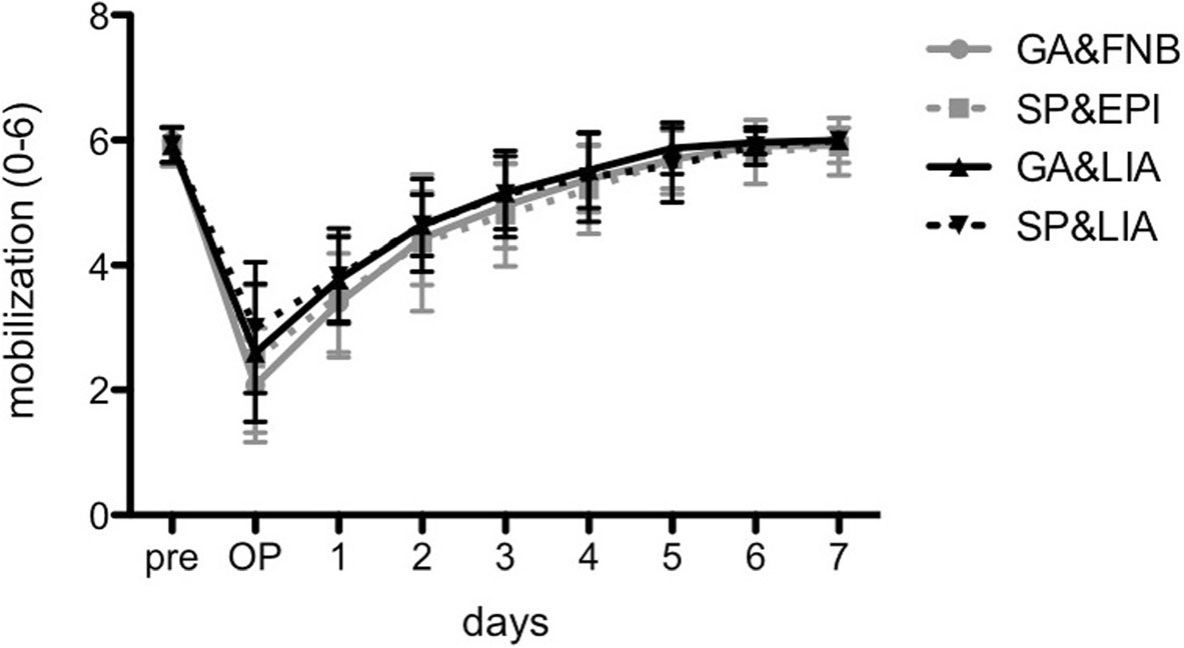
The grade of mobilization (0 to 6) revealed a gradual increase after surgery until postoperative day 7

The grade of mobilization (0 to 6) revealed a gradual increase after surgery (Fig. 3). Hereby, all groups reached their base level of mobilization at day 6. At the day of surgery and postoperative day 1, GA&FNB presented a significantly lower grade of mobilization (2.1 ± 0.9 and 3.4 ± 0.8, respectively) compared to SP&LIA (3.0 ± 1.0 and 3.8 ± 0.7; *p* = 0.003 and *p* = 0.007, respectively) and GA&LIA (3.8 ± 0.7; *p* = 0.005; only at day 1). From postoperative day 2 on, no further significant differences among the groups could be detected (*p* > 0.05).

### Muscle strength

**Fig. 4:**
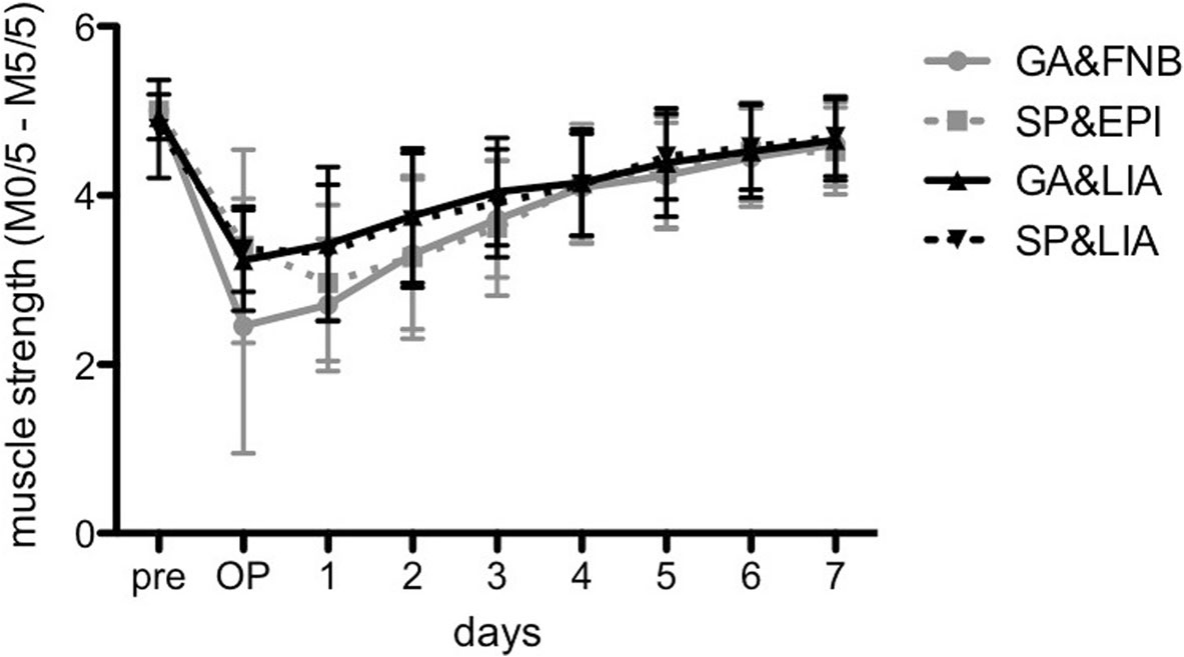
The grade of muscle strength (M0/5 to M5/5) after surgery until postoperative day 7 is shown

The results of the muscle strength (Fig. 4) according to the British medical research council (M0/5-M5/5) showed a homogeneous increase after surgery with reaching strength against resistance (values ≥4) in all groups from day 3 on. Compared to the LIA groups, GA&FNB revealed statistically significant reduced muscle strength at day 1 (3.4 ± 0.9 and 3.3 ± 0.8 vs. 2.7 ± 0.8 each; *p* < 0.001 and *p* = 0.001, respectively) and day 2 (3.8 ± 0.8 and 3.7 ± 0.8 vs. 3.3 ± 0.9 each; *p* = 0.011 and *p* = 0.039, respectively); at day 3 only GA&LIA showed higher muscle strength compared to GA&FNB (4.0 ± 0.6 vs. 3.7 ± 0.7; *p* = 0.019). Additionally, the values of SP&EPI were significantly lower compared to GA&LIA from day 1 to 3 (2.9 ± 0.9 vs.3.4 ± 0.9, *p* = 0.042; 3.3 ± 1.0 vs. 3.8 ± 0.8, *p* = 0.026 and 3.6 ± 0.8 vs. 4.0 ± 0.6, *p* = 0.024, respectively).

### Range of motion

**Fig. 5:**
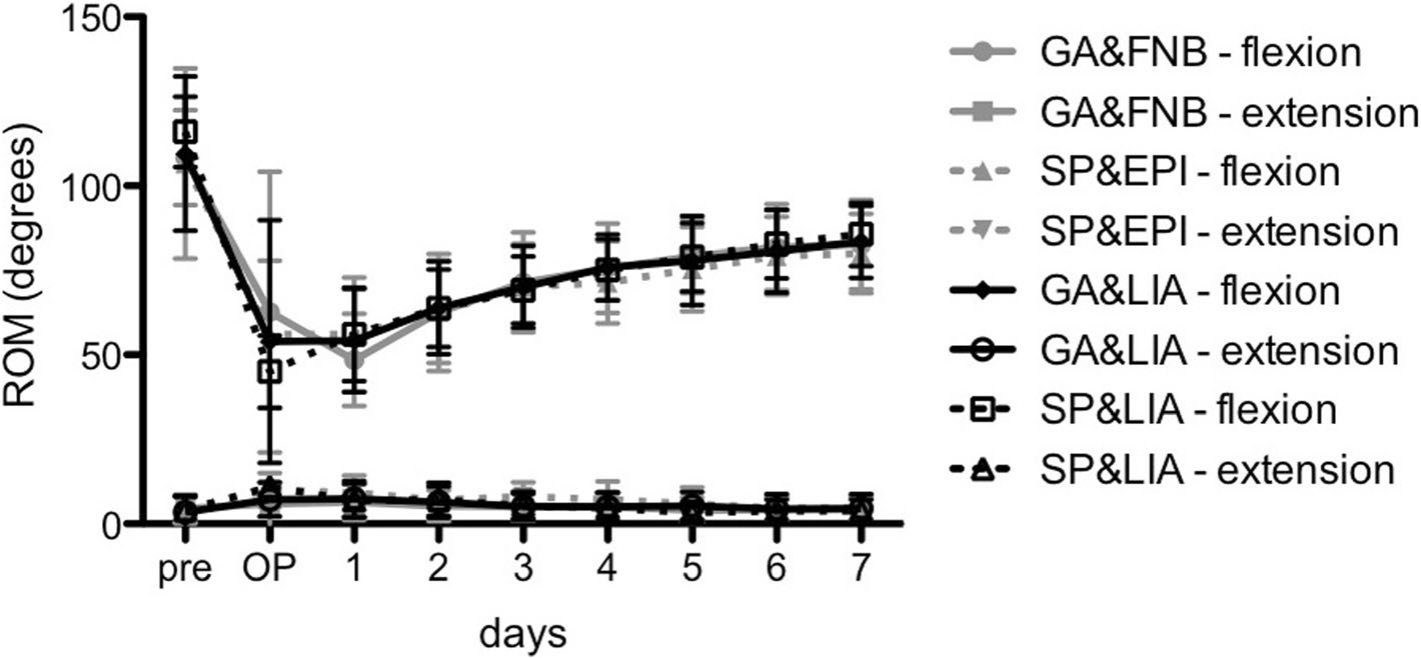
Degrees of range of motion (flexion and extension) of all groups from preoperative day to postoperative day 7 are presented

The mean range of motion (flexion and extension of the knee joint) was comparable and not different among the groups (*p* > 0.05, Fig. 5) with the exception of SP&LIA which demonstrated a higher flexion at day 1 (56.1° ± 13.9°) compared to GA&FNB (48.5° ± 13.7°; p = 0.001). From postoperative day 1, flexion gradually increased while extension decreased. Considering all groups, 80° of flexion was reached on day 6.

## Discussion

The aim of the LIA technique is to release the therapeutic effect by blocking pain conduction at its origin. Here, the distribution and the quantity of the injected local anesthetics are of great importance. Therefore, our injection was performed according to a standardized protocol to systematically reach every part of the knee joint’s surrounding tissue. In an anatomic study, the anatomical spread of a LIA used in TKA was examined to identify the nerve structures reached by the injected fluid [19]. The results supported the positive clinical outcomes of the LIA techniques by visualization of good infiltration of the majority of nerves supplying the knee. Only in the lower popliteal fossa less solution could be detected. However, in particular, the posterior part of the knee is the most frequent area, where patients have pain postoperatively. By use of peripheral nerve blocks, this area is generally addressed by a SNB. Some clinical trials suggested that a SNB could improve postoperative analgesia when added to a FNB in patients undergoing TKA [5, 6]. To address this posterior knee pain, we regularly perform a FNB combined with a single shot SNB or we infiltrated the posterior articular capsule thoroughly with ropivacaine (0.2%) in the LIA groups. Thereby, it is important to start with the posterior LIA injection before implantation of the inlay in order to have enough access to the posterior aspect.

In literature, most trials reported reduced pain and opioid requirements in the early (< 48 h) postoperative period with LIA compared with placebo or no injection [11–13]. In a meta-analysis, Keijsers et al. showed an average decrease in pain scores at rest of 12.3% and a decrease in opioid consumption of 14.8% compared to placebo at 24 h after surgery [20]. No significant differences were found between FNB (comparable with GA&FNB) and epidural catheters (SP&EPI) for TKA in terms of pain (at rest and with activity) in the early postoperative period [3, 7]. However, the side effects (e.g. hypotension or urinary retention) were higher with epidural catheters, which could not be detected within the first postoperative days in our study. Data comparing LIA with continuous epidural analgesia are limited and favor LIA over continuous epidural analgesia, particularly due the side-effects and early mobilization [16–18].

Compared to (peripheral) analgesic catheters, LIA generally achieves comparable results with regard to postoperative pain [21–23]. A recent systematic review and meta-analysis comparing FNB and LIA after total knee arthroplasty did not reveal any differences in morphine consumption and pain scores on postoperative day 1 [22]. These findings were in concordance with our results of GA&FNB and GA&LIA showing no significant differences in NRS scores at rest and with activity. However, in the very early postoperative period (≤6 h), LIA seems to be able to achieve a better reduction in pain [14, 15]. In our group, interestingly, at the day of surgery the catheter-based groups showed a significantly lower dose (on average 42%) of hydromorphone compared to the LIA groups (*p* < 0.001). Furthermore, the spinal anesthesia group with an epidural catheter (SP&EPI) revealed an up to 58% lower dose of hydromorphone from the day of surgery until day 3 compared to all other groups. Consistent with our results, Harsten et al. described a significantly reduced pain score with spinal anesthesia within the first 12 h in contrast to general anesthesia while both anesthetic procedures seem to generate the same overall pain ratings [24]. This is clinically relevant as a reduced opioid consumption consequently minimizes opioid-related adverse events. Furthermore, patients with a higher pain level preoperatively may rather benefit from spinal anesthesia and an epidural catheter.

But most mentioned studies just focused on pain relief and did not consider further functional aspects including mobilization, muscle strength or range of motion. In contrast to LIA groups, patients of GA&FNB were less likely to be able to stand in front of their bed on the day of surgery or even start to ambulate at postoperative day 1; an effect attributed to possible residual muscular weakness from the two peripheral nerve blocks. These findings were supported by Safa et al., who also demonstrated a significantly minor mobilization in the FNB/SNB group compared to LIA at day 1 [25]. However, there were no falls or short-term complication in the following days and functional recovery was comparable after 7 days among all groups. But the improved mobilization and significant higher muscle strength in the early postoperative period allow earlier intensive rehabilitation therapies and reduce especially in elderly patients the risks of a prolonged immediate postoperative immobilization (e.g. deep venous thrombosis, pulmonary infection). McDonald et al. also showed this trend in a randomized controlled trial comparing spinal anesthesia with epidural analgesia and LIA [23].

A common concern of the LIA technique is the potential toxic side effects, which might be elicited by a joint and tissue infiltration of local anesthetics like ropivacaine. Knudsen et al. defined the toxic threshold in arterial samples for unbound concentration of ropivacaine to be 0.56 μg/ml and for maximum total concentration to be 4.3 μg/ml [26]. A pharmacokinetic study with periarticular (single shot) infiltration of 400 mg ropivacaine (0.2%) after TKA revealed that the maximum unbound ropivacaine amounted to a concentration of 0.13 μg/ml and, therefore, remained far below the toxic threshold [27]. Although we did not analyze the plasma levels of ropivacaine in our study, we did not run the risk reaching the toxic threshold at any time by dose of 200 mg ropivacaine (0.2%).

There are some limitations that pertain to that study. The length of hospital stay was not a measured outcome of this study, however, all of our TKA patients followed a clinical pathway with standardized discharge criteria. This included the ability to ambulate and negotiate stairs with an assistive device, independent activities of daily living as well as knowledge of how to progress and continue an independent exercise program with an established plan for discharge physiotherapy. Furthermore, due to its retrospective design, the study was not blinded or randomized, which may have introduced reporting bias. The choice of anesthesia by the patient might also have induced some selection bias, although the group characteristics appeared to be identical among the four groups. Although it was a retrospective investigation, the strengths of the study include a large number of patients managed according to clear inclusion and exclusion criteria. The surgical and anesthetic procedures followed a consistent standard-treatment protocol in the same hospital by the same surgeons with extensive surgical experience in the treatment of total knee arthroplasties and its concomitant analgesic procedures.

## Conclusions

In conclusion, the findings from this study suggest an advantage of LIA in the early postoperative periods in terms of mobilization and muscle strength. Range of motion was comparable among all groups. Pain control was also similar in all groups, while the use of rescue pain medication was significantly lower with SP&EPI compared to all other groups within the first postoperative days. Thus, clinicians should consider the risk-to-benefit ratio for each case individually.

## Abbreviations

FNB: Femoral nerve block
GA&FNB: General anesthesia and femoral nerve block (+sciatic nerve block)
GA&LIA: General anesthesia and local infiltration analgesia
LIA: Local infiltration analgesia
NRS: Numeric rating scale
OP: Operation (day of surgery)
PCIA: Patient-controlled intravenous analgesia
SNB: Sciatic nerve block
SP&EPI: Spinal anesthesia and epidural catheter
SP&LIA: Spinal anesthesia and local infiltration analgesia
TKA: Total knee arthroplasty
